# Two-Year Clinical Performance of Ultra-Thin No-Prep Veneers from 5Y-TZP Zirconia: A Retrospective Study

**DOI:** 10.3390/bioengineering12090976

**Published:** 2025-09-15

**Authors:** Katarzyna Taraszkiewicz-Sulik, Patryk Wiśniewski, Edyta Cywoniuk, Teresa Sierpińska

**Affiliations:** 1 Department of Prosthetic Dentistry, Medical University of Białystok, 15-089 Bialystok, Poland; teresa.sierpinska@umb.edu.pl; 2 Bio Dental Clinic Dr. Katarzyna Taraszkiewicz-Sulik, DDS, PhD, General Partnership—Private Practice, 15-453 Bialystok, Poland; scanbiodental@gmail.com; 3 Department of Periodontal and Oral Mucosa Diseases, Medical University of Białystok, 15-089 Bialystok, Poland; patryk.wisniewski@umb.edu.pl

**Keywords:** zirconia veneers, no-prep, Prettau^®^ Skin, minimally invasive dentistry, adhesive cementation, biofunctional restorations

## Abstract

Objective: This retrospective study aimed to evaluate the two-year clinical performance of ultra-thin, no-prep Prettau^®^ Skin zirconia veneers placed in the anterior region of the maxilla and mandible. Materials and Methods: This single-cohort retrospective series did not include a conventional control group. A total of 201 veneers (Prettau^®^ Skin, 5Y-TZP zirconia) were placed in the anterior maxilla and mandible. Veneers were air-abraded with 50 µm Al_2_O_3_ (0.25 MPa, ~10 mm, 20 s) and bonded using an MDP-containing adhesive (Tokuyama Bond, Tokuyama Dental, Japan) and dual-cure resin cement (Estecem II, Tokuyama Dental, Japan) following enamel etching with 37% H_3_PO_4_ (Etching Gel, Cerkamed, Poland). Clinical performance was assessed using the modified FDI criteria after two years. Results: At 24 months, no debonding events were recorded. The survival rate was 99.5% (95% CI: 97.3–99.9). Fracture rate was 0.5% (95% CI: 0.1–2.8). Most veneers received “very good” scores for surface luster (81.6%, 95% CI: 75.6–86.4), color match (96.0%, 95% CI: 92.0–98.0), marginal adaptation (84.1%, 95% CI: 78.3–88.6), and anatomical form (100%, 95% CI: 98.1–100). Periodontal response was rated as “very good” or “good” in 90.0% (95% CI: 85.4–93.4) of cases. Patient satisfaction remained consistently high (100%, 95% CI: 98.1–100). Conclusions: Ultra-thin, no-prep Prettau^®^ Skin zirconia veneers show favorable short-term clinical outcomes, offering excellent esthetic results, mechanical stability, and biological compatibility. These findings support their use as a minimally invasive option in anterior restorative dentistry. However, further long-term studies are needed to confirm their durability and compare outcomes with conventional veneer techniques.

## 1. Introduction

Modern esthetic dentistry increasingly follows the principle of minimal invasiveness, aiming to achieve optimal outcomes with minimal interference to hard dental tissues [1]. Veneers represent one of the most established approaches within this philosophy and can, in selected cases, replace conventional prosthetic restorations. Depending on the clinical situation, preparation techniques range from no-prep and minimal-prep to standard preparation, with the latter involving greater enamel reduction [2,3]. The non-prep approach is particularly attractive because it preserves enamel integrity, avoids anesthesia, and minimizes the risk of pulpal or structural complications, but is applicable only in carefully selected cases such as mild discolorations, diastemas, or minor incisal defects [4,5,6]. Careful patient selection and precise digital planning are therefore essential to achieving predictable outcomes.

Alongside this trend, increasing attention has been given to zirconium dioxide (ZrO_2_) for ultra-thin restorations. Zirconia offers high strength, wear resistance, and excellent biocompatibility [7], but its lack of a glassy phase historically limited adhesive applications [8]. Advances in bonding strategies—particularly the use of phosphate monomers (e.g., 10-MDP) combined with surface conditioning techniques such as alumina sandblasting or activation—have made stable resin adhesion more predictable [9,10,11]. Nevertheless, zirconia’s chemically inert surface still renders long-term adhesion technique-sensitive. Clinical studies suggest that while ultrathin zirconia veneers can achieve reliable short- to mid-term retention [12], debonding remains the most common complication in resin-bonded fixed dental prostheses [13,14]. For conventionally cemented zirconia crowns, loss of retention has also been reported, though often linked to preparation geometry, cement selection, and operator-dependent factors rather than the ceramic itself [15]. More recently, experimental multi-acid etching protocols (e.g., Zircos-E) have been introduced to chemically modify zirconia surfaces and further enhance resin bonding [16,17]. These developments underline that the adhesive potential of zirconia is evolving but still requires careful clinical validation.

**Figure 1:**
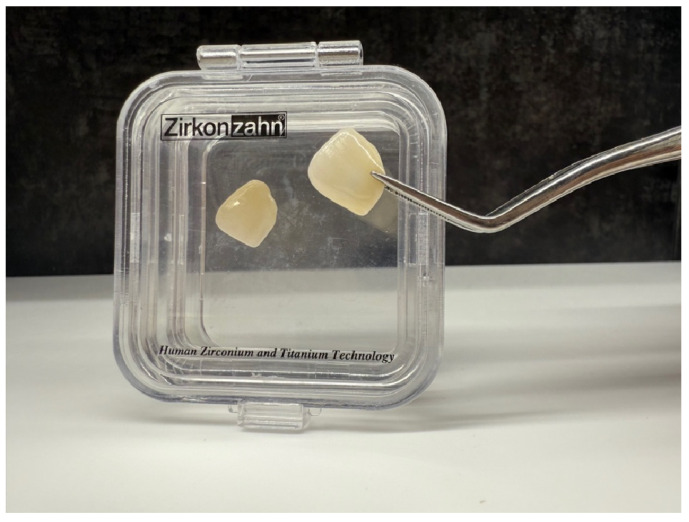
Prettau^®^ Skin (Zirkonzahn) veneer placed on tooth 21—case from the authors’ own material.

Parallel to these advances in adhesion, material innovations have expanded the indications for no-prep approaches. Highly translucent monolithic zirconia, such as Prettau^®^ CAD/CAM veneers, combines the mechanical durability of zirconia with improved optical properties, achieving esthetics closer to natural dentition. Digital workflows enable precise adaptation, while a minimum thickness of 0.2 mm allows truly no-prep application, in line with the principles of biologically conservative dentistry (Figure 1). This development bridges the gap between minimally invasive protocols and the functional reliability of zirconia-based ceramics.

Despite these advances, clinical evidence on ultra-thin, no-prep zirconia veneers remains scarce. While feldspathic and lithium disilicate veneers are well documented, medium- to long-term data on monolithic zirconia alternatives are lacking. Moreover, few studies have assessed biological and patient-centered outcomes in addition to technical performance, further limiting the available evidence base. This study therefore aimed to provide a two-year retrospective clinical evaluation of Prettau^®^ no-prep zirconia veneers, focusing on survival, complications, and functional, biological, and patient-centered outcomes.

## 2. Materials and Methods

### 2.1. Study Characteristics, Participants and Design

The present study was based solely on the analysis of medical records of adult patients treated at the dental office “Bio Dental Clinic sp.j.” in Białystok, Poland, between October 2022 and June 2023. This retrospective analysis of existing clinical records complied with the Declaration of Helsinki. Under local regulations, prior ethics committee approval was not required for anonymized chart reviews. All patients provided written informed consent for treatment and the use of anonymized data.

All treatments were carried out by the same clinician. According to the documentation, patients had primarily sought esthetic dental procedures, including improvement of tooth shape and color, closure of diastemas, and restoration of anterior teeth following trauma.

The exclusion criteria, as specified in the records, included poor oral hygiene, active periodontal disease, bruxism, temporomandibular joint disorders, and the need for tooth preparation for prosthetic restoration. Cases with existing labial restorations or dentin exposure at bonding surfaces were also excluded to ensure enamel-only bonding in line with the no-prep protocol.

In total, 201 veneers were placed on anterior teeth—158 on maxillary teeth and 43 on mandibular teeth—in 30 patients (22 females and 8 males), aged between 26 and 40 years (mean age 34.7), as documented in the medical files. In instances where the records were incomplete, missing data were verified and supplemented using photographic documentation and follow-up visit records, ensuring a complete dataset for analysis.

### 2.2. Pre-Treatment and Laboratory Procedures

**Figure 2:**
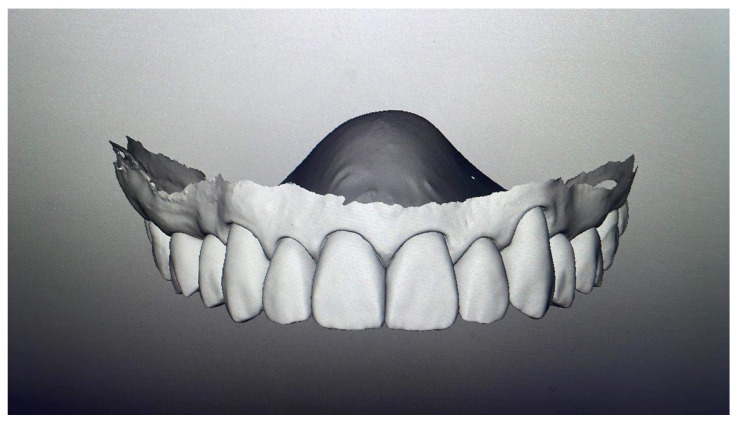
Virtual mock-up created using 3Shape Trios Design Studio^®^ software, version 2022.1 (3Shape, Copenhagen, Denmark).

Diagnostic procedures included a comprehensive dental and radiological examination. Patient documentation was performed using intraoral scans, dental models, photographs, and video recordings that captured various facial and intraoral views at rest, natural smile, and full smile. Intraoral scanning (TRIOS^®^ Intraoral Scanner, 3Shape, Copenhagen, Denmark) was used to register the three-dimensional dental situation and for subsequent analysis. The data obtained were processed with 3Shape Trios Design Studio^®^ software, and a virtual mock-up was created. If the esthetics, phonetics, and function of the virtual mock-up were satisfactory and accepted by the patient, the order for the final restoration was sent to the laboratory (Figure 2).

### 2.3. Clinical Try-In and Luting Procedure

After evaluating the final restorations on 3D-printed models, the clinical try-in was carried out on hydrated teeth under standardized lighting. Fit and marginal integrity were verified under surgical loupes, and shade selection was confirmed chairside. Where needed, a neutral glycerin-based try-in gel (Variolink Esthetic Try-In Paste, Ivoclar Vivadent AG, Schaan, Liechtenstein) was used to simulate the optical effect of the resin cement prior to final seating. Upon successful verification of fit and color, the veneers were adhesively cemented. Teeth isolation was achieved using a rubber dam (NicTone Rubber Dam, NicTone, Szczecin, Poland) and a retraction cord size #0000 (Retraction Cord size 0000, Cerkamed, Stalowa Wola, Poland) using a single-cord technique. The teeth were then cleaned with a brush and fluoride-free polishing paste (Cleanic Paste, Kerr, Orange, CA, USA). The enamel surface was etched with 37% phosphoric acid gel for 15 s (Etching Gel, Cerkamed, Stalowa Wola, Poland), thoroughly rinsed for 15 s, and dried. The inner surface of the zirconia veneer was sandblasted under the following parameters: 50 µm Al2O3 abrasive (Alublast, Henry Schein, Melville, NY, USA), 0.25 MPa pressure (2.0 bar), 10 mm distance, 20 s duration.

The restorations were then placed in an ultrasonic cleaner with isopropyl alcohol (Isopropyl Alcohol, Cerkamed, Stalowa Wola, Poland) for 2 min and thoroughly dried. Subsequently, a bonding agent (Tokuyama Bond, Tokuyama Dental, Tokyo, Japan) was applied in one layer for approximately 20 s according to the manufacturer’s instructions and air-dried until solvent evaporation, followed by light curing for 10 s with a 1200 mW/cm^2^ LED lamp (Elipar DeepCure-S, 3M ESPE, St. Paul, MN, USA).

The same bonding agent was applied and rubbed into the tooth surface for 20 s, then air-dried and light cured as described above. A thin layer of resin cement (Estecem II, Tokuyama Dental, Tokyo, Japan) was applied to the inner veneer surface. After seating the veneer, excess cement was removed from interproximal spaces with dental floss and from the gingival margin area using a micro-brush. Initial light curing was performed for 2 s to facilitate excess removal, followed by final polymerization for 20 s.

### 2.4. Occlusal Adjustment and Polishing

**Figure 3:**
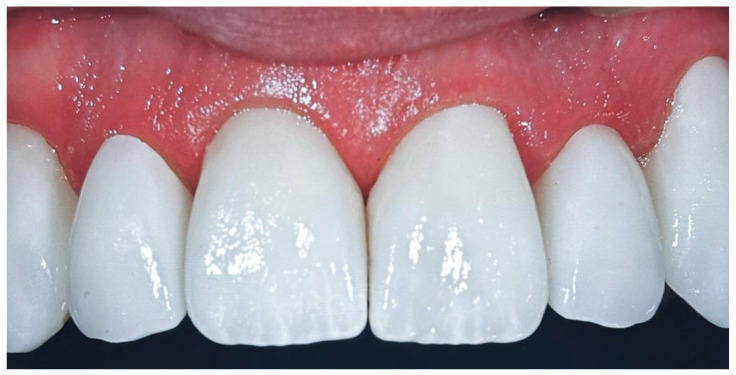
Immediate postoperative condition following the cementation of veneers on teeth 13–23.

During the final finishing stage, any residual excess material was removed from the cervical and proximal areas. If removal was difficult, manual gradual polishing was performed using a sequential protocol: 15 µm diamond bur (Komet # 316-016 Bud FG), diamond strips (Soft-Lex Finishing Strips, 3M ESPE, Seefeld, Germany), zirconia polishing rubber (CeraGlaze Diamond Polishing Rubber P332, Kerr Rotary GmbH, Germany), and ceramic polishing pastes (Enamel Shiny Diamond Polishing Paste ZIRCO, Micerdent, Poland). The prepared tooth and the external veneer surface were also polished. Static and dynamic occlusion was checked using 40 µm articulating paper (Bausch Arti-Check^®^ 40 µm, Bausch GmbH, Germany). When restorations altered the intermaxillary relations (e.g., increased vertical dimension or changes in lateral and protrusive guidance), minor adjustments with a diamond bur followed by final polishing were performed (Figure 3).

### 2.5. Evaluation

Clinical outcomes were assessed for each veneer (*n* = 201) based on entries in the patients’ medical records, using the FDI World Dental Federation criteria [18], which evaluate three main parameters: esthetics, function, and biological compatibility. Each parameter was rated on a five-point FDI scale by two independent clinicians during follow-up visits. Any discrepancies were resolved by consensus.

The esthetic assessment, as recorded, included observations on color stability, shape, translucency, and marginal adaptation, based on intraoral examinations and photographic documentation. Evaluations were performed by two clinicians, and any discrepancies noted in the records were resolved by consensus.

Functional evaluation documented in the records included restoration stability, structural integrity, adequacy of contact points, and occlusal contacts verified using articulating paper.

Biological criteria, as noted in the charts, comprised the presence of secondary caries (based on visual and tactile examination), periodontal status assessed by bleeding on probing (BoP) using a CP-15 periodontal probe (Hu-Friedy, Chicago, IL, USA), pulp vitality assessed with ethyl chloride testing (Pulp Spray, Cerkamed, Stalowa Wola, Poland), and the overall oral health status.

Statistical analysis of the collected data was performed using Statistica version 13.0 (StatSoft Inc., Tulsa, OK, USA). Descriptive statistics are reported with 95% confidence intervals (Wilson method) for proportions.

## 3. Results

A total of 201 Prettau^®^ Skin veneers were evaluated two years after cementation using modified FDI World Dental Federation criteria, covering esthetics, function, and biological response. Ratings were recorded on a five-point scale: 1—very good, 2—good, 3—satisfactory, 4—unsatisfactory, 5—poor.

### 3.1. Esthetic Parameters

Most esthetic parameters received the highest ratings. Surface luster was rated very good in 81.59% and good in 18.41% of cases, closely matching the patients’ natural enamel. Surface color match was very good in 96.02% and good in 3.98%, while marginal color match received very good ratings in 96.52% and good in 3.48%. Color and translucency stability were very good in 79.60% and good in 20.40%. The anatomical form was rated very good in 100% of cases (Table 1).

### 3.2. Functional Parameters

Fractures of veneers occurred in only one case (0.68%), involving marginal chipping, which was successfully polished. In other cases, fracture rating was very good (96.62%) or good (2.70%). Marginal adaptation was very good in 84.08%, good in 15.42%, and satisfactory in 0.50% of cases. Occlusal contours and wear were very good in all cases (100%), as were proximal contacts. Patient satisfaction regarding esthetics and function was uniformly very good (100%) (Table 2).

### 3.3. Biological Parameters

Pulp vitality and absence of hypersensitivity were rated very good in 98.51% and good in 1.49%. No carious or non-carious lesions were detected (100% very good). No fractures of abutment teeth were observed. Periodontal response was rated very good in 51.24%, good in 38.81%, satisfactory in 6.97%, and unsatisfactory in 2.98% (gingivitis grade I to III). Oral mucosa and general oral health were very good in all cases (100%) (Table 3).

## 4. Discussion

Our results confirm the high effectiveness of ultra-thin, no-prep Prettau^®^ Skin zirconia veneers, aligning with the growing trend toward minimally invasive esthetic dentistry. The excellent esthetic outcomes and preservation of tooth tissue demonstrate the advantages of using ultrathin ceramic veneers without enamel preparation.

Our findings correspond with those of De Angelis et al. (2023) and Śmielak et al. (2024), who also reported high esthetics and durability for non-prep feldspathic ceramic veneers [6,19]. However, unlike feldspathic ceramics, our study highlights the superior mechanical strength of zirconia, consistent with Koutayas et al. (2009) [7]. We confirmed the efficacy of surface treatment by sandblasting zirconia and using 10-MDP-containing adhesive cements [8,9].

This protocol enabled durable bonding with no microfractures or discoloration, as supported by Souza et al. (2018) [11]. Adhesive cementation to enamel has been reported to yield better outcomes than bonding to enamel and dentin, resulting in fewer complications such as debonding or secondary caries [20,21]. Our no-prep approach enabled bonding exclusively to enamel, thus enhancing adhesion reliability.

Compared to Marquillier et al. (2018), who reported higher translucency of feldspathic ceramics, Prettau^®^ Skin veneers demonstrated satisfactory esthetics, suggesting that modern manufacturing techniques and optimal veneer thickness minimize visual differences [18].

Biological stability findings agree with Breschi et al. (2008), who emphasized the importance of oral hygiene for maintaining periodontal health around ceramic restorations [22]. No significant deterioration in periodontal tissues was observed, indicating a safe clinical profile. The gingivitis observed in a small fraction of patients was likely related to biofilm control rather than the veneers themselves.

When compared with other ceramic systems, our two-year results are consistent with findings from randomized and long-term clinical studies. Nguyen et al. (2025) conducted a randomized controlled trial comparing acid-etched zirconia veneers bonded with MDP-containing adhesives to lithium disilicate veneers, reporting no significant differences between the two groups across esthetic, functional, and biological parameters after one year. Both materials achieved high survival rates, with failure modes differing—zirconia veneers tended to debond but could be successfully rebonded, whereas lithium disilicate veneers were more prone to fracture upon debonding. These findings further support the clinical viability of zirconia veneers as a durable and esthetic alternative to lithium disilicate [23].

Similiary, the three-year RCT by Fawakhiri et al. (2023) demonstrated no significant differences between zirconia and lithium disilicate veneers regarding esthetic, functional, and biological outcomes, though lithium disilicate displayed slightly higher translucency [24]. Similarly, the meta-analysis by Klein et al. (2024) reported a pooled survival rate of 96.8% for lithium disilicate veneers at 10.4 years, closely matching our short-term observations [25]. Feldspathic and leucite-reinforced veneers showed slightly lower survival, with long-term studies reporting rates of 93–96% and higher complication rates, particularly fractures and chipping. Beier et al. (2012) observed survival of 94.4% at five years, 93.5% at ten years, and 82.9% at twenty years for silicate veneers, while Layton et al. (2012) estimated a ten-year survival rate of 96.0% for feldspathic veneers, and Guess et al. (2008) reported 97.5% survival at five years for overlap veneers [26,27,28]. These findings indicate that zirconia veneers can achieve early outcomes comparable to lithium disilicate and feldspathic ceramics, though their long-term performance remains to be validated.

Material characteristics may further explain these differences. Zirconia exhibits higher flexural strength (800–1200 MPa) and fracture toughness (6–8 MPa·m½) compared with lithium disilicate (360–440 MPa; 2.5–3.0 MPa·m½), which may enhance resistance to crack propagation in thin designs or high-stress conditions [29]. Conversely, lithium disilicate offers superior translucency, making it advantageous in highly esthetic cases [30]. Thus, the choice of material should be individualized, balancing mechanical durability with esthetic demands.

The paucity of literature on zirconia no-prep veneers highlights the innovative nature of our research and the need for further long-term clinical evaluations to confirm their efficacy and longevity.

It is important to note several significant limitations of our study. First, the relatively short follow-up period precludes the assessment of veneer durability over a longer timeframe, especially regarding mechanical wear during daily function. Second, the limited number of patients in the study reduces the ability to generalize the results to a broader population. Third, the absence of a control group receiving conventionally prepared veneers restricts direct comparisons of the efficacy between the two methods. Additionally, we did not systematically record or analyze potential confounding factors such as individual oral hygiene levels, occlusal characteristics, or the presence of parafunctional activities. While bruxism and active periodontal disease were part of the exclusion criteria, unmeasured differences in daily hygiene and functional habits may still have influenced the outcomes. Finally, the use of only basic clinical evaluation methods without advanced imaging or microscopic analyses limits the scope of information on the biological impact of the veneers.

Taken together, these limitations mean that the validity of our findings should be interpreted with caution, particularly regarding their generalizability to other patient groups and longer follow-up periods. Nevertheless, the results provide valuable preliminary evidence on the short-term performance of ultra-thin zirconia veneers and highlight the need for future prospective, controlled, and long-term clinical trials.

## 5. Conclusions

Prettau^®^ Skin non-prep zirconia veneers represent a clinically valuable option for patients seeking highly esthetic outcomes while preserving natural tooth structure. Their combination of minimal invasiveness, predictable bonding, and favorable biological response makes them a promising choice in contemporary restorative dentistry. From a clinical perspective, this approach allows practitioners to achieve long-lasting esthetics with a reduced risk of biological complications.

Nevertheless, robust evidence from larger cohorts and long-term comparative studies with established veneer materials remains essential to fully validate the durability and effectiveness of this treatment modality.

## Figures and Tables

**Table 1:** Esthetic evaluation using FDI criteria.

Parameter	1 (Very Good)	95% CI (Very Good)	2 (Good)	3 (Satisfactory)	4 (Unsatisfactory)	5 (Poor)
Surface luster	164 (81.59%)	75.6–86.4	37 (18.41%)	0 (0%)	0 (0%)	0 (0%)
Surface color match	193 (96.02%)	92.0–98.0	8 (3.98%)	0 (0%)	0 (0%)	0 (0%)
Marginal color match	194 (96.52%)	92.6–98.4	7 (3.48%)	0 (0%)	0 (0%)	0 (0%)
Color and translucency stability	160 (79.60%)	73.5–84.7	41 (20.40%)	0 (0%)	0 (0%)	0 (0%)
Anatomical form	201 (100%)	98.1–100.0	0 (0%)	0 (0%)	0 (0%)	0 (0%)

**Table 2:** Functional evaluation using FDI criteria.

Parameter	1 (Very Good)	95% CI (Very Good)	2 (Good)	3 (Satisfactory)	4 (Unsatisfactory)	5 (Poor)
Fractures (marginal/other surfaces)	194 (96.52%)	92.6–98.4	5 (2.49%)	2 (0.99%)	0 (0%)	0 (0%)
Marginal adaptation	169 (84.08%)	78.3–88.6	31 (15.42%)	1 (0.50%)	0 (0%)	0 (0%)
Occlusal contour and wear	201 (100%)	98.1–100.0	0 (0%)	0 (0%)	0 (0%)	0 (0%)
Proximal contacts	201 (100%)	98.1–100.0	0 (0%)	0 (0%)	0 (0%)	0 (0%)
Patient’s view	201 (100%)	98.1–100.0	0 (0%)	0 (0%)	0 (0%)	0 (0%)

**Table 3:** Biological evaluation using FDI criteria.

Parameter	1 (Very Good)	95% CI (Very Good)	2 (Good)	3 (Satisfactory)	4 (Unsatisfactory)	5 (Poor)
Vitality/pulp and hypersensitivity	198 (98.51%)	95.5–99.5	3 (1.49%)	0 (0%)	0 (0%)	0 (0%)
Caries/non-carious lesions	201 (100%)	98.1–100.0	0 (0%)	0 (0%)	0 (0%)	0 (0%)
Fractures of tooth	201 (100%)	98.1–100.0	0 (0%)	0 (0%)	0 (0%)	0 (0%)
Periodontal response	103 (51.24%)	44.4–58.0	78 (38.81%)	14 (6.97%)	6 (2.98%)	0 (0%)
Oral mucosa	201 (100%)	98.1–100.0	0 (0%)	0 (0%)	0 (0%)	0 (0%)
General oral health	201 (100%)	98.1–100.0	0 (0%)	0 (0%)	0 (0%)	0 (0%)

## Data Availability

Data available on request due to restrictions privacy or ethical reasons.
